# Third-party disability: An analysis of speech-language therapists’ experiences in adult dysphagia management in South Africa

**DOI:** 10.4102/ajod.v15i0.1841

**Published:** 2026-01-31

**Authors:** Kim Coutts, Daniella Meyerowitz

**Affiliations:** 1 Department of Speech Pathology, Faculty of Humanities, University of the Witwatersrand, Johannesburg, South Africa

**Keywords:** dysphagia, third-party disability, third-party caregivers, counselling, ICF framework, South Africa

## Abstract

**Background:**

Dysphagia, a swallowing impairment, has left people in need of assistance and care. Therefore, the lives of caregivers to those individuals with dysphagia change drastically. There is scarce research about the difficulties experienced by caregivers in caring for their loved ones with dysphagia, a phenomenon known as third-party disability (TPD).

**Objectives:**

This study explored Speech Language Therapists’ (SLTs’) experiences with caregivers when managing adult patients living with dysphagia in South Africa.

**Method:**

This study made use of a qualitative approach through an online survey with an optional follow-up interview using an adapted framework of the International Classification of Functioning, Disability and Health (ICF) model. Thirteen participants took part in the survey and two completed the follow-up online interview. Data were analysed using a top-down thematic analysis approach.

**Results:**

Third-party caregivers receive counselling at various times with different content because of the lack of a standardised counselling protocol. Furthermore, using the ICF framework, the article identified that environmental, and contextual factors contribute to TPD. However, not all ICF components were applicable, and additional relevant factors were not captured.

**Conclusion:**

Understanding SLTs’ experiences in managing TPD in the adult dysphagia population was achieved.

**Contribution:**

The study contributes to literature regarding TPD of adult patients living with dysphagia and has captured the role of the SLT in managing TPD in diverse settings across South Africa.

## Introduction

As a result of strokes, neurological degenerative conditions, respiratory distress and head and neck cancers, many individuals are left with a swallowing impairment known as dysphagia (Kim & Kahrilas 2019). Not only does dysphagia affect an individual’s ability to swallow, but it also affects their daily living activities.

Moreover, they lose their independence for the long-term future and may rely on caregivers to attend to them to ensure safety (Namasivayam-MacDonald & Shune 2020). By looking after their loved ones, caregivers’ lives have changed to become a person responsible for taking care of the wants and needs of the individual with dysphagia. This change in lifestyle indirectly impairs the caregiver and aligns with a new phenomenon known as third-party disability (Namasivayam-MacDonald & Shune 2020).

While there is growing recognition of this phenomenon, research on third-party disability (TPD) in the context of dysphagia remains limited, as much of the literature has focused primarily on the impact of dysphagia on those directly affected (Mach et al. 2019). Furthermore, the evident gap between Global North and South-based research will be further explored.

Notably, Namasivayam-MacDonald and Shune (2020) have examined the effects of caregiver burden on ageing parents. Nund et al. (2014, 2015) described TPD in the management of dysphagia related to head and neck cancer, including non-surgical interventions. Grawburg et al. (2013) further explored TPD in relation to aphasia. Collectively, these Global North based studies underscore that although TPD has received some attention, the specific impact of swallowing disorders on caregiver burden remains underexplored. There is a paucity of research investigating TPD as it relates to dysphagia more broadly. The lack of research regarding TPD in relation to dysphagia is a significant gap, as eating and adapting how people eat profoundly affect multiple dimensions of both the individual’s and the caregiver’s lives. In addition, from the various articles found above one may conclude that most of the TPD research has been conducted and published in the Global North, limiting the relevance of these findings to the Global South, where resource constraints, linguistic diversity, and cultural differences shape caregiving in unique ways (Howell 2019).

Many of the studies from the Global North have applied the International Classification of Functioning, Disability and Health (ICF) model as a framework to conceptualise TPD (Nund et al. 2015). While the ICF model highlights the interplay between body function and structure, activity and participation and environmental and personal factors of the person with the disability only, it can be altered to include TPD caregivers. However, the ICF model does not fully capture the in depth lived experiences, burdens or adaptations of the caregivers. Therefore, this model cannot be relied upon exclusively to represent the holistic troubles experienced by the caregivers.

**FIGURE 1:**
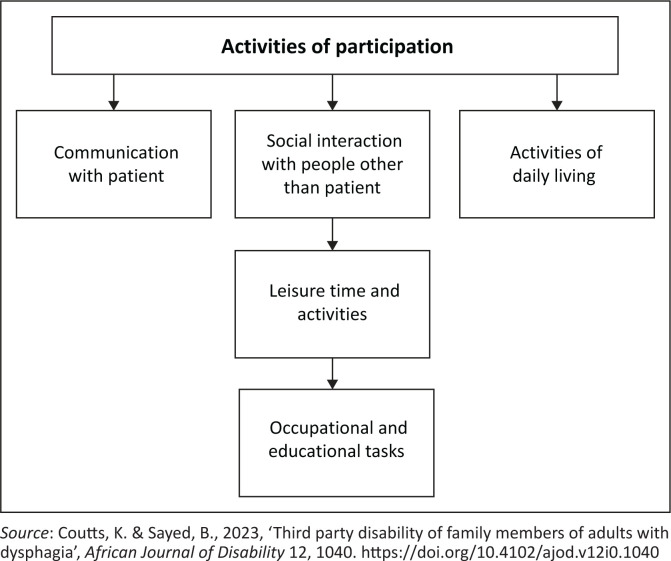
International Classification of Functioning, Disability and Health subthemes.

To address this limitation, Coutts and Sayed (2023) developed an alternative framework to complement the ICF. In this alternative framework, the authors created various subthemes under the typical themes incorporated in the ICF model. Furthermore, they elaborated on how all the themes are interconnected, an aspect that is not explicit in the original ICF model. Instead of focusing on the activity and participation of the person with the disability only, the caregiver was targeted, as seen in Figure 1. This framework informed the data collection and analysis in this study.

It is understood that Speech Language Therapists (SLTs) play a vital role in the management of dysphagia patients (Carnaby & Harenberg 2013). The SLTs are responsible for providing the patient with manoeuvres and strategies as well as modifying diets to improve swallowing functions and ensure safe swallowing (Carnaby & Harenberg 2013). While the main form of therapy is given to the patient themselves, adaptation of diets as a result of dysphagia affect the patient’s caregivers profoundly (Namasivayam-MacDonald & Shune 2020). Additionally, it is within the Speech Language Pathologist’s scope of practice to counsel patients (Sekhon et al. 2019). It is evident that counselling needs to be given to adults of dysphagia pertaining to their disease and its effect on their life course. However, further counselling must be given to the family and caregivers of the dysphagic individual in teaching them how to care for their loved one and highlighting the necessary intervention to combat the burdens experienced by these TPD caregivers. The extent to which this is done, is not known. By using the adapted form of the ICF model, the SLT is able to get a clear understanding of the experienced difficulties of the TPD caregivers and provide adequate counselling to these caregivers.

Due to South Africa (SA)’s limited resources resulting from majority low-to-middle income households, the country has a complex sociocultural and environmental context. This is further highlighted by the evident multiculturalism leading to varying perspectives of dysphagia. It is therefore essential that SLTs conceptualise and integrate TPD into their practice. This study aimed to explore SLTs’ experiences when managing TPD of adult patients with dysphagia.

## Research methods and design

A qualitative exploratory research design was conducted as it was a design that described an area that was scarcely researched (Hunter, McCallum & Howes 2019). Speech Language Therapists’ perspectives of TPD were scarcely researched and were therefore an appropriate example. However, some quantitative aspects were incorporated throughout the research report.

The proposed sample size of this research article comprised 13 participants. The purpose of this sample size was to ensure that saturation of information was attained (Braun & Clarke 2021). Both snowball sampling and purposive sampling were used in this research article where implied consent was obtained. The participants were required to click a link which was sent in April 2024, which took them to a Google Form created survey. The survey consisted of a mixture of multiple choice questions and long answered questions.

At the end of the survey, there was an opportunity to tick for an optional Zoom follow-up interview where a consent form was given as well as a space for the participant to upload their email address for them to be contacted. The purpose of the follow-up interview was for expansion of the ideas contained in the survey including the cultural and caregiver attitudinal differences experienced by the Speech Therapists (STs). Two participants took part in the optional follow-up interview. The inclusion and exclusion criteria are shown in Table 1.

**TABLE 1:** Inclusion and exclusion criteria.

Inclusion criteria	Exclusion criteria
SASHLA-registered SLTs but not limited to SASHLA members.	Family members
SLTs who have been exposed to people affected by adult patients with dysphagia.	Patients with dysphagia
SLTs who have access to the internet and who can operate Zoom.	Student STs

SLT, Speech Language Therapist; SASHLA, South African Speech Language Hearing Association; ST, Speech Therapist.

The data were analysed using the Braun and Clarke’s approach to thematic analysis (Braun & Clarke 2017). In their approach, the positivist psychological framework was used to identify, analyse and interpret qualitative information (Braun & Clarke 2017). Furthermore, the data were organised using a top-down approach. The report first focused on the main argument and thereafter explained the findings using smaller subthemes. The data were analysed using two theoretical frameworks. Firstly, the theoretical framework used in the top-down approach was based on components included in the ICF model (Nund et al. 2015). Secondly, aspects from the adapted ICF framework were incorporated for analysis purposes, as used by Coutts and Sayed (2023). Lastly, because of the use of both qualitative research and quantitative aspects, some information in the research report was analysed using descriptive statistics.

It was essential to establish trustworthiness, rigour, credibility, dependability and generalisation to enable valid and reliable research (Amankwaa 2016). Credibility and reliability were attained through the process of use-of-self (Shufutinsky 2020). The researcher employed the use-of-self approach through reflexivity to ensure personal biases were not ensued upon the analysis. Rigour refers to being precise, diligent, and exact (Johnson, Adkins & Chauvin 2020). Furthermore, consistent questions were used for all participants in both the survey and the follow-up interviews. Lastly, thick descriptions were used and incorporated in the findings including quotations and examples to enhance reliability (Amankwaa 2016). Rigour was adhered to through the thorough and careful collection of data, verbatim quoting and member checking (Amankwaa 2016). Next, dependability was achieved to ensure that the data could be repeated and that the findings were consistent (Amankwaa 2016). Dependability was attained through a process known as inquiry audit, the process in which an external researcher who was not involved in the data collection examined the process and the product of the research study (Amankwaa 2016).

### Ethical considerations

Ethical clearance to conduct this study was obtained from the University of Witwatersrand non-medical research committee (No. STA_2024_07). Various ethical considerations were used throughout the data collection and analysis:

- **Informed consent:** Participant were required to sign a consent form for the online survey and additional optional online interview.
- **Anonymity:** Efforts to ensure anonymity were used through the omission of participant’s names and differential information.
- **Confidentiality:** No information was shared as the data were only seen by the authors.
- **Data management:** All data were stored electronically in a password-protected document and device that only the authors had access to. This research will be kept for 6 years for reanalysis purposes.
- **Vulnerability management:** Data costs were informed prior to the completion of the survey and online interview. Furthermore, if the participants displayed behaviours of emotional distress they were referred to Ms Pabolla Lepota at the Emthonjeni Centre Clinic to receive necessary help from treating psychologists. No participants required these services.

## Results

### Participant demographics

The participants who completed the online survey comprised of 13 Health Professions Council of South Africa (HPCSA)-registered SLTs. It was observed that seven participants have had less than 5 years of experience, four have had more than 5 years and two have been practising for more than 10 years. Only Participant 2 worked in a public hospital while the rest either worked in private hospitals or private practice.

### Themes that emerged

The results of the data collected in the online survey and interview stemmed from a larger study. Only the following significant findings will be presented using various themes and subthemes (Table 2).

**TABLE 2:** Themes and subthemes emerged from the collected data.

Theme	Subtheme
1. Knowledge of TPD	-
2. Counselling	2.1 Counselling – Time, Type and Setting 2.2 Content of Information Given
3. ICF factors	3.1 Activities and Participation 3.2 Environmental Factors
4. Attitude of loved ones	-

ICF, International Classification of Functioning, Disability and Health; TPD, third-party disability.

#### Theme 1: Knowledge of third-party disability

In the survey, nine participants were able to accurately define TPD. The overarching definition was summarised by one participant:

‘Ways that a family member might be impacted by their loved one’s health condition.’ (Participant 2, public hospital, 2 years experience, Gauteng)

Furthermore, the participants were asked to identify TPD caregivers. Using descriptive statistics, it may be concluded that nine participants indicated caregivers, seven included family, three included spouses and nurses and two said parents, as mentioned by by one participant:

‘[*C*]aregivers, spouses and family members’. (Participant 5, private hospital, 3 years experience, Gauteng)

One participant included siblings and Participant 3 said that ‘friends in a caring role’ should also be included in this category. However, while most participants have a working understanding of TPD, many do not. In the survey, only two out of 13 participants indicated that they were unfamiliar with the term ‘TPD’.

In addition, two participants identified that they understood the term TPD, but gave inaccurate definitions.

The following two quotes highlight this misunderstanding:

‘An additional party involved in the patient’s care who requires knowledge of the patient’s condition/disability.’ (Participant 11, private hospital, 8 years experience, Gauteng)

‘A disability caused by something or someone.’ (Participant 13, private hospital, 3 years experience, Gauteng)

While it is important to understand the term TPD, it is equally important to understand who is suffering from TPD. Therefore, the participants were asked to identify who they thought were TPD caregivers. Among the correct TPD caregivers detected, two participants (Participant 13 and Participant 7) mistakenly identified nurses, *medical aids and case managers* and *kitchen staff*. From the results, one cannot fully generalise that all SLTs understand TPD and its appropriate included caregivers.

#### Theme 2: Counselling

**Subtheme 2.1: Counselling – Time, type and setting:** From the answers of the online survey, various counselling times, forms of counselling and settings were highlighted. With regard to time of counselling, the participants were asked to determine at which point during the rehabilitation process they counsel TPD caregivers. Participants 2, 4, 9, 10 and 13 counsel immediately after seeing the patient for the first time. Participants 1 and 8 counsel after conducting an assessment with the patient and Participants 5, 6 and 11 counsel only after starting management. However, three additional participants indicated that they counsel at alternate times as mentioned by Participant 7, ‘after each session’.

Therefore, the times that families are counselled vary between SLTs. In addition, two types of counselling may be given to TPD caregivers, namely informational counselling and personal adjustment counselling. Participants 3, 5, 6, 8, 9, 11, and 12 used the former while Participant 10 used the latter. The remaining four participants used ‘both informational counselling and personal adjustment counselling’.

Lastly, the participants stated in which setting they counsel their patients. Participants 2, 7, 8, 11 and 13 counselled in a one-on-one setting while Participants 1, 4, 5, 9, 10 and 12, counselled both the patient and the TPD caregivers simultaneously. It must be observed that Participant 6 counselled the TPD caregivers using a ‘multidisciplinary team’ and Participant 3 used ‘any of the above depending on the context’. Based on the results, there is no standardised counselling time, counselling type and counselling setting used by the participants.

Additionally, while Speech Language Pathologists (SLPs) counsel their patients and their appropriate TPD caregivers, the universities do not equip the therapists to counsel. Two participants indicated in an online interview that the university syllabus did not help them with their counselling skills:

‘I don’t think we had a specific model for counselling.’ (Participant 5, private hospital, 3 years experience, Gauteng)

Therefore, they learnt to counsel TPD caregivers either at their practical sites, through observing their supervisors or during their community service year:

‘When we were at the practical sites, the supervisors would give advice about counselling families and patients on their diagnosis.’ (Participant 5, private hospital, 3 years experience, Gauteng)

This has implications for differing content given and caregivers receiving the counselling.

**FIGURE 2:**
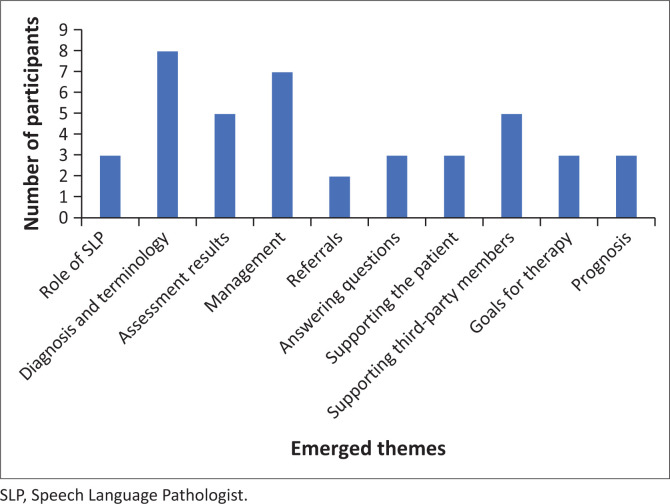
Counselling content of information given to third-party caregivers.

**Subtheme 2.2: Content of information given:** The results of the content of information given to the TPD caregivers may be seen in the graph in Figure 2.

From the graph shown in Figure 2, three participants explained their role as SLTs:

‘The role of the Speech-Language Therapist and an explanation of dysphagia and Speech-Language fallouts.’ (Participant 11, private hospital, 8 years experience, Gauteng)

Eight participants explained the patient’s diagnosis and its accompanying terminology. While five participants explained the assessment results, seven discussed the management plan for the patient. Two participants spoke about referrals and three answered any proposed questions. Moreover, three participants explained ways in which the TPD caregivers help the patient:

‘I do my best to provide them with support regarding the possible impacts to their life.’ (Participant 4, private practice, 10 years experience, Gauteng)

Four participants highlighted ways to help the wellbeing of the TPD caregivers. Finally, three participants highlighted the specific and targeted goals for the rehabilitation process:

‘I explain a bit more about the goals of therapy.’ (Participant 10, private hospital, 5 years experience, Gauteng)

#### Theme 3: International Classification of Functioning, Disability and Health factors

**Subtheme 3.1: Activity and participation:** This section of the model focuses on the specific activities of TPD caregivers that will be limited as a result of caring for their loved one with dysphagia. For this, the Coutts and Sayed (2023)’s adapted version of the ICF for TPD was used.

**Activities of daily living:** The results stated that TPD caregivers struggle to perform typical daily activities following the diagnosis of their loved ones with dysphagia. Three participants stated that TPD caregivers need to change the consistency of their loved one’s diet through the use of thickeners or blenders to ensure safe feeding. In an interview with a participant, they recounted an experience with a TPD member where she exclaimed:

‘How am I going to cook for this person.’ (Participant 10, private hospital, 5 years experience, Gauteng)

Furthermore, three additional participants stated that TPD caregivers struggle to help the family member once they have been discharged from the hospital as highlighted by Participant 1 as the *inability to manage*. These two activities of daily living such as preparing meals and managing the home, change drastically because of their loved one being diagnosed with dysphagia.

**Leisure:** Participant 13 stated that TPD caregivers ask, ‘if they can still go to restaurants’, following their loved one’s dysphagia diagnosis. Because of their loved one having dysphagia, they may only be able to eat certain foods or consistencies that are inaccessible at restaurants.

**Subtheme 3.2: Environmental factors:** All participants indicated that finances contributed to TPD. By taking care of a loved one with dysphagia, from the SLT perspectives, they found that caregivers are subjected to additional financial burdens. Two participants stated that the costs of alternative forms of feeding such as Percutaneous Endoscopic Gastrostomy (PEG) tubes and PEG feeds are expensive:

‘[*T*]he aspects such as PEG tubes would need to be replaced and the costs of it.’ (Participant 12, private practice, 3 years experience, Gauteng)

In addition, six participants stated that eating utensils and cooking apparatus such as blenders will need to be bought to ensure safe consistencies and eating practices as evidenced by one comment:

‘[*F*]unding a blender for making a puree.’ (Participant 7, private hospital, 3 years experience, KwaZulu Natal)

For families to attend therapy sessions, two participants said they will need to pay for public transport, one said they need to pay for the therapy session, three stated the costs of medical aid, and two participants highlighted that third-party caregivers pay for frailty centres or caregivers to assist their loved ones:

‘[*M*]ainly the costs of caregivers and frail care facilities.’ (Participant 10, private hospital, 5 years experience, Gauteng)

Additionally, to take their loved one to therapy, one participant highlighted that the third-party caregivers will not be able to go to work thereby losing further income. This may cause major financial unrest within the family as the caregiver will either quit their job to provide for the patient with dysphagia or the caregiver will need to pay for a full-time carer at a great expense to the family.

#### Theme 4: Attitude of loved ones

When caring for a patient with dysphagia, the attitude of the TPD caregivers may either contribute to or alleviate their experienced TPD. Both individuals who participated in an online interview stated that a positive attitude with committed attendance to the therapy sessions, persistent acknowledgement of understanding, practice and an overall enthusiastic approach, the caregiver burden experienced by TPD caregivers may be diminished. One participant stated that you can determine a positive caregiver attitude when:

‘[*T*]hey bring carers, when they are there, when they are asking questions, even if it is not the right questions.’ (Participant 5, private hospital, 3 years experience, Gauteng)

However, there are times when TPD caregivers do not always have a positive attitude towards caring for their loved one. A TPD member exclaimed:

‘When I go home, I am going to give her food and I am going to try to feed her because I feel like you guys didn’t do enough.’ (Participant 5, private hospital, 3 years experience, Gauteng)

By not feeding her loved one correctly, she is putting the individual in danger of aspiration and even death.

## Discussion

### Participants’ demographics

Thirteen participants living in SA took part in this research report. With scarce published articles highlighting the influence of TPD in the Global South, this article contributes to the Southern database, but much more information is needed to close the gap in research. From the results, it was concluded that seven of the 13 participants had under 5 years of experience, representing a younger, newer-perspective group. Interestingly, despite the younger participant population, evidence suggests that TPD and counselling were not included in their curriculum, stemming from recent teachings (WITS n.d.). This may limit the services given to the TPD caregivers because of a limitation in the SLTs’ clinical readiness and ethical management. Therefore, there is a need for curriculum review at an undergraduate level to close these evident gaps. Furthermore, only one participant indicated that they practised in a public vicinity. Hence, the data may be biased towards patient experiences in private practice and may not consider the TPD contextual factors experienced in the public sector, where over 80% of health care users make use of public services (Pillay et al. 2020).

### Knowledge of third-party disability

Majority of the participants were able to accurately define TPD and identify its appropriate TPD caregivers.

However, while people may think they understand the term and its associated members, they do not fully know what the term means, as evidenced by two participants. The lack of knowledge could be influenced by gaps in the curriculum but cannot be generalised to all participants. Nurses, kitchen staff, medical aid and case managers are not TPD caregivers as they are either providing a service or are not directly impacted by the patient’s disability. Inaccurate identification of TPD caregivers has negative implications in the attempt to mitigate TPD. Even if SLTs are actively trying to diminish the disability felt by the TPD caregivers, the wrong people will be targeted (Grawburg et al. 2013). Furthermore, from the results of the article, a lack of knowledge could influence the quality of counselling and services provided to TPD. Regular trainings, interdisciplinary learning and reflective supervision should be provided to working SLTs who did not have TPD training in their curriculum to facilitate knowledge and enable best practice post qualification.

### Counselling

From the results obtained, it was clear that counselling of TPD caregivers occurs at different times, with varying types of services provided and often with contrasting information. Optimal counselling practice from SLTs towards patients and their family members should occur regularly throughout the rehabilitation process (Sekhon et al. 2019), as the needs of both patients and caregivers evolve across different stages of recovery (Sekhon et al. 2023). A significant finding was that key aspects such as prognosis, therapy goals, patient support, referrals, clarification of the SLT’s role, and opportunities for questions were frequently omitted. The absence of these discussions not only limits caregivers’ understanding of dysphagia but also raises ethical concerns, as caregivers may be left unsupported in contexts where they are already overwhelmed. This highlights the relational responsibility of SLTs to provide consistent, transparent, and empathetic communication that acknowledges the emotional and practical burdens carried by caregivers.

The results further suggested that group counselling, bringing together patients, caregivers, and potentially members of the multidisciplinary team (MDT), may be an optimal approach (De Schrijver, Leye & Merckx 2016).

Such an approach could enhance shared understanding and provide relational support by normalising caregiver experiences. However, its feasibility requires more critical interrogation for the complex South African healthcare sector. In the private sector, group counselling may be more achievable because of resource availability, while in the public sector, systemic barriers such as limited MDT availability, misaligned visiting and working hours, and high caseloads (Malakoane et al. 2020) present significant challenges. These contextual realities underscore the need to balance the ethical significance of offering relationally supportive counselling with the practical constraints of different healthcare settings.

The lack of established counselling protocols for TPD caregivers emerged as a critical gap. Without standardised, evidence-based guidelines, counselling practices risk being inconsistent, ethically variable, and insufficiently responsive to caregiver needs. This finding has important practice implications, particularly in the South African public healthcare context, where resource constraints heighten the need for structured, equitable approaches. Further research is therefore required to develop and evaluate counselling protocols that are both ethically grounded and contextually feasible, ensuring that SLTs can provide consistent, relationally attuned, and sustainable support to TPD caregivers.

### International Classification of Functioning, Disability and Health factors

The results revealed three emergent ICF factors contributing to TPD taken from the adapted model by Coutts and Sayed (2023): financial status, activities of daily living, and leisure. Financial challenges were largely because of the high cost of feeding materials, with alternative feeding methods being particularly expensive – posing a burden even in the private sector (Malakoane et al. 2020). Third-party disability caregivers also need to take time off work to attend therapy sessions and hospital visits with their loved one. For many, the loved one with dysphagia may have been working before their diagnosis, which would lead to a loss in income. South Africa has a population of 12 000 homeless individuals (South African Government 2022). In some households, both the patient loses their job as a result of their diagnosis and its associated disabilities, as well as the third-party member to care for their loved ones. While the questions focused on ICF implementation, no specifics regarding its unique application in the South African context were explored. This is a limitation as cultural adaptions and local relevance is extremely important and should have been further explored. The predominance of participants working in private practice may have limited consideration of the broader public-sector population, highlighting a gap for further research.

Coutts and Sayed (2023)’s adapted ICF model includes subthemes within ‘activity and participation’, two of which – activities of daily living and leisure – were identified by participants as relevant to TPD. Although this study viewed the model from the SLT’s perspective rather than TPD caregivers themselves, the adapted model’s additional subthemes offer a more holistic representation and are recommended for use in future TPD considerations. Broader research including public healthcare contexts is warranted.

### Attitudes of the caregiver

Both positive and negative experiences of caregivers’ attitudes towards their loved ones were highlighted in the study. Many caregivers demonstrated eagerness to engage in the rehabilitation process, often asking questions and seeking guidance. This aligns with findings by Mori et al. (2024), who found that caregivers are more motivated to provide care after consulting with professionals. When TPD caregivers adopt a constructive attitude, they tend to view caregiving not as a burden but as a meaningful contribution to their loved one’s recovery, which can reduce their own sense of strain. However, the results also revealed more complex relational dynamics that extend beyond a simple positive–negative dichotomy. Caregivers may experience uncertainty, simultaneously wanting to support their loved one while feeling overwhelmed by competing responsibilities. Role strain was evident where caregivers struggled to balance employment, family obligations, and the demands of dysphagia care. Moreover, cultural norms and expectations, particularly within the South African context, where extended family structures and caregiving roles are often gendered, shaped how caregiving responsibilities were perceived and enacted. These factors highlight the need for a more nuanced understanding of caregiver attitudes and their impact on rehabilitation.

It is important to recognise that while caregiver attitudes can either facilitate or hinder treatment, the way SLTs respond to these dynamics is equally critical (Shafer, Haley & Jacks 2023). In the absence of formal counselling protocols, SLTs often rely on professional judgement, ad hoc strategies, or informal support from colleagues to navigate situations where caregivers are disengaged, resistant, or ambivalent. This raises ethical and relational challenges, as SLTs may feel underprepared to address the psychosocial dimensions of caregiver burden. Support structures such as peer supervision, case discussions, and referral pathways to psychologists or social workers are sometimes available, but their accessibility varies significantly between private and public healthcare sectors.

The call for MDT collaboration is therefore appropriate, as social workers and psychologists can provide expertise in managing caregiver distress and relational strain (Fareo 2015). However, collaboration is constrained by scope-of-practice boundaries, limited staffing, and systemic barriers such as high caseloads, misaligned schedules, and resource shortages in the public sector. These challenges underscore the need for clearer interprofessional protocols and systemic support to ensure that caregiver counselling is both ethically sound and practically feasible. Future research should therefore not only focus on developing standardised, evidence-based counselling protocols for TPD caregivers but also critically examine how such protocols can be implemented within the realities of South African healthcare systems.

Hence, the insights gained from these findings form the foundation for the research limitations and implications discussed next.

### Research limitations

#### Limited generalisability

The research report highlights the TPD of adult patients with dysphagia. Because of the specificity of a dysphagia diagnosis, it may not be generalisable to TPD of patients with other diagnoses.

#### Sample size

The sample size of this research is small. Thirteen participants answered the online survey. Furthermore, only two participants answered the follow-up online interview. With a small sample size, in depth knowledge, and varied perspectives of TPD of adult patients with dysphagia from the perspectives of SLTs could not be attained. In addition, there is a need for more participants who work in the public sector.

#### A lack of pilot study

This research report did not use a pilot study because of the small sample size. However, from the findings, it is understood that the tools used to collect data were reliable and yielded effective results. However, because of the small sample size, this research could be termed a ‘pilot study’ in which future studies may use it as base.

#### Use of a survey

This research report made use of a survey to collect data. While it is an appropriate way to gather information, participants may answer the survey multiple times. This may affect the reliability of the data collection.

### Implications

Several important implications emerged from conducting this research. Firstly, there is a clear need for a standardised protocol to guide counselling for third-party individuals caring for a loved one with dysphagia. Such a protocol should outline the type of counselling provided, the setting in which it occurs, and the specific content to be communicated. Secondly, future research should involve a larger sample size to gain a more comprehensive understanding of SLT practices and the variation in management approaches across different provinces and countries. It is also recommended that more SLTs working in the public sector be included in subsequent studies to ensure diverse perspectives are captured. Finally, further investigation is needed into the undergraduate curriculum related to counselling, including how it is taught and what practical training opportunities are provided to strengthen students’ counselling skills.

## Conclusion

This study aimed to examine TPD in adult patients with dysphagia in South Africa, as perceived by Speech-Language Therapists. Specifically, it explored SLTs’ knowledge of TPD and their identification of relevant third-party caregivers and analysed how they apply both the ICF framework and an adapted version when considering these caregivers. Findings indicate the need for further research to develop a standardised protocol for counselling third-party caregivers in dysphagia care. In addition to mapping professional knowledge and practice, the study offers insight into the personal experiences of South African SLTs working with third-party caregivers affected by a patient’s swallowing disorder.
